# Reforms for financial protection schemes towards universal health coverage, Senegal

**DOI:** 10.2471/BLT.19.239665

**Published:** 2019-12-05

**Authors:** Bocar Mamadou Daff, Serigne Diouf, Elhadji Sala Madior Diop, Yukichi Mano, Ryota Nakamura, Mouhamed Mahi Sy, Makoto Tobe, Shotaro Togawa, Mor Ngom

**Affiliations:** a Agence Nationale de la Couverture Maladie Universelle, Dakar, Senegal.; b Graduate School of Economics, Hitotsubashi University, Tokyo, Japan.; c Hitotsubashi Institute for Advanced Study, Hitotsubashi University, 2-1, Naka, Kunitachi, Tokyo, 186-8601, Japan.; d Japan International Cooperation Agency, Tokyo, Japan.

## Abstract

Advancing the public health insurance system is one of the key strategies of the Senegalese government for achieving universal health coverage. In 2013, the government launched a universal health financial protection programme, *la Couverture Maladie Universelle*. One of the programme’s aims was to establish a community-based health insurance scheme for the people in the informal sector, who were largely uninsured before 2013. The scheme provides coverage through non-profit community-based organizations and by the end of 2016, 676 organizations had been established across the country. However, the organizations are facing challenges, such as low enrolment rates and low portability of the benefit package. To address the challenges and to improve the governance and operations of the community-based health insurance scheme, the government has since 2018 planned and partly implemented two major reforms. The first reform involves a series of institutional reorganizations to raise the risk pool. These reorganizations consist of transferring the risk pooling and part of the insurance management from the individual organizations to the departmental unions, and transferring the operation and financial responsibility of the free health-care initiatives for vulnerable population to the community-based scheme. The second reform is the introduction of an integrated management information system for efficient and effective data management and operations of the scheme. Here we discuss the current progress and plans for future development of the community-based health insurance scheme, as well as discussing the challenges the government should address in striving towards universal health coverage in the country.

## Résumé

Faire progresser le système public d'assurance maladie est l'une des principales stratégies du gouvernement sénégalais, qui ambitionne de rendre les soins de santé accessibles à tous. En 2013, le gouvernement a lancé un programme de protection financière global en la matière, la Couverture Maladie Universelle. L'un des objectifs de ce programme consistait à établir un régime communautaire d'assurance maladie pour les personnes appartenant au secteur informel, encore largement non assurées auparavant. Ce régime fournit une couverture par le biais d'organismes communautaires sans but lucratif. Fin 2016, 676 organismes de ce type avaient été créés aux quatre coins du pays. Néanmoins, ces organismes sont confrontés à des défis tels que le faible taux d'inscription et la transférabilité réduite de la gamme d'avantages sociaux. Pour y remédier, mais aussi pour améliorer la gouvernance et les opérations du régime communautaire d'assurance maladie, le gouvernement a planifié et partiellement appliqué deux réformes d'envergure depuis 2018. La première implique une série de réorganisations institutionnelles afin d'accroître la mutualisation des risques. Ces réorganisations consistent à transférer la mutualisation des risques et une partie de la gestion de l'assurance de chacun des organismes vers les unions départementales, et à confier au régime communautaire la responsabilité financière et la mise en œuvre des initiatives destinées à prodiguer des soins de santé aux populations les plus vulnérables. La seconde prévoit l'introduction d'un système de gestion intégrée de l'information afin d'administrer les données et les opérations plus rapidement et avec davantage d'efficacité. Dans ce document, nous évoquons les progrès actuels et les projets de développement futur du régime communautaire d'assurance maladie. Nous traitons également des défis que le gouvernement doit relever, ainsi que des efforts déployés pour offrir une couverture maladie universelle à l'ensemble du territoire.

## Resumen

La promoción del sistema público de seguro médico es una de las estrategias clave del Gobierno senegalés para lograr la cobertura sanitaria universal. En 2013, el gobierno lanzó un programa de protección financiera universal de la salud, la Couverture Maladie Universelle. Uno de los objetivos del programa era establecer un sistema comunitario de seguro médico para las personas del sector informal, que en su mayoría no tenían seguro antes de 2013. El sistema proporciona cobertura a través de organizaciones comunitarias sin fines de lucro y, a finales de 2016, se habían establecido 676 organizaciones en todo el país. Sin embargo, las organizaciones se enfrentan a desafíos, como las bajas tasas de inscripción y la baja portabilidad del paquete de prestaciones. Para hacer frente a los desafíos y mejorar la gobernanza y el funcionamiento del sistema comunitario de seguro médico, desde 2018 el Gobierno ha planificado y aplicado parcialmente dos reformas importantes. La primera reforma implica una serie de reorganizaciones institucionales para elevar las fuentes de riesgo. Estas reorganizaciones consisten en la transferencia de la mancomunación de riesgos y parte de la gestión de los seguros de las distintas organizaciones a los sindicatos departamentales, y en la transferencia de la operación y la responsabilidad financiera de las iniciativas de atención gratuita de la salud para la población vulnerable al sistema comunitario. La segunda reforma consiste en la introducción de un sistema integrado de información de gestión para una gestión de datos y un funcionamiento eficientes y efectivos del sistema. Aquí se discuten los avances actuales y los planes para el desarrollo futuro del sistema comunitario de seguro médico, así como los desafíos que el gobierno debe abordar en su lucha por lograr la cobertura sanitaria universal en el país.

## ملخص

إن الارتقاء بنظام التأمين الصحي الشعبي هو أحد الاستراتيجيات الرئيسية للحكومة السنغالية لتحقيق التغطية الصحية الشاملة. أطلقت الحكومة في عام 2013 برنامج الحماية المالية الصحية الشامل، تحت اسم *la Couverture Maladie Universelle * . كان أحد أهداف البرنامج هو تأسيس مخطط مجتمعي للتأمين الصحي للأشخاص في القطاع غير الرسمي، والذين كان يفتقرون إلى حد كبير إلى التأمين قبل عام 2013. ويوفر المخطط التغطية من خلال المنظمات المجتمعية غير الهادفة للربح وبحلول نهاية عام 2016، تم إنشاء 676 مؤسسة في أنحاء الدولة. ومع ذلك، فإن المنظمات تواجه تحديات، مثل انخفاض معدلات الالتحاق وانخفاض قابلية نقل حزمة المزايا. لمواجهة التحديات وتحسين الحوكمة وعمليات مخطط التأمين الصحي المجتمعي، قامت الحكومة منذ عام 2018 بالتخطيط والتنفيذ الجزئي لاثنين من الإصلاحات الرئيسية. يتضمن الإصلاح الأول سلسلة من عمليات إعادة التنظيم المؤسسية لرفع مجموعة المخاطر. تتألف عمليات إعادة التنظيم تلك من نقل تجميع المخاطر، وجزء من إدارة التأمين من المؤسسات الفردية إلى اتحادات الإدارات، ونقل التشغيل والمسؤولية المالية لمبادرات الرعاية الصحية المجانية للسكان المعرضين للخطر إلى مخطط مجتمعي. كان الإصلاح الثاني هو إدخال نظام متكامل للمعلومات الإدارية، بهدف تحقيق الكفاءة والفعالية لإدارة لبيانات وعمليات المخطط. نحن نناقش هنا التقدم الحالي وخطط التنمية المستقبلية لمخطط التأمين الصحي المجتمعي، بالإضافة إلى مناقشة التحديات التي يجب أن تواجهها الحكومة في سعيها نحن تحقيق التغطية الصحية الشاملة في البلاد.

## 摘要

推进公共医保体系是塞内加尔政府实现全民健康覆盖的关键战略之一。2013 年，政府发起了一项全民医疗财政保障计划，即 la Couverture Maladie Universelle。该计划其中的一个目标是为大部分 2013 年以前未投保的非正式部门人口建立一个以社区为基础的医保计划。该计划通过非营利性社区组织进行覆盖，截至 2016 年底，全国已建立 676 个组织。然而，这些组织现在正面临着诸如入学率低和福利方案不可大范围应用等难题。为了解决上述难题，改善社区医保计划的管理和运作，政府从 2018 年开始就计划并部分实施了两项重大改革。第一项改革包括一系列组织重组，加强风险分担。这些重组包括将风险分担和部分保险管理从个别组织转移到部门工会，并将针对弱势群体的免费医疗计划的运作和财务责任转移到社区计划。第二项改革是引入综合管理信息系统，以实现高效的数据管理和计划运营。在此，我们讨论了当前社区医保计划的进展和未来发展计划，以及政府在全国范围内实现全民健康覆盖可能会应对的挑战。

## Резюме

Развитие государственной системы медицинского страхования — одна из ключевых стратегий Правительства Сенегала для достижения всеобщего охвата услугами здравоохранения. В 2013 году правительство запустило универсальную программу финансовой защиты в сфере здравоохранения *la Couverture Maladie Universelle*. Одной из целей программы было создание схемы медицинского страхования на уровне общин для населения, занятого в неформальном секторе, которое в основном не было охвачено страхованием до 2013 года. Схема предусматривает обеспечение страхового покрытия за счет некоммерческих организаций на уровне общин, количество которых к концу 2016 года составило 676. Тем не менее эти организации сталкиваются с такими проблемами, как низкий уровень охвата и малая преемственность пакета страховых услуг. Чтобы решить эти проблемы и совершенствовать управление и работу схем медицинского страхования на уровне общин, правительство запланировало и частично осуществило две главные реформы в период с 2018 года. Первая реформа предусматривает ряд реорганизаций на государственном уровне с целью создания страхового пула. Такая реорганизация предусматривает передачу страхового пула и части управления страхованием от отдельных организаций в объединения на уровне департаментов, а также включение управления и финансовой ответственности за реализацию инициатив по предоставлению услуг бесплатного медицинского обслуживания наиболее уязвимым группам населения в схему медицинского страхования на уровне общин. Вторая реформа представляет собой внедрение единой информационной системы управления для эффективного и действенного управления данными и действиями в рамках данной схемы. В статье обсуждается текущее состояние и планы по дальнейшему развитию схемы медицинского страхования на уровне общин, а также обсуждаются проблемы, которые следует решить правительству для обеспечения всеобщего охвата услугами здравоохранения в стране.

## Introduction

Target 3.8 of the sustainable development goal 3 is to achieve universal health coverage (UHC), including financial risk protection, access to quality essential health-care services and access to safe, effective, quality and affordable essential medicines and vaccines for all.^1^ In the strive to achieve UHC and target 3.8, many countries are reforming their public health insurance system.^2^^–^^4^ For example, the Kenyan government reformed their insurance system and increased enrolment from 8.40 million people in June 2011 to 27.20 million in June 2018.^5^^,^^6^ In Senegal, only 2.68 million people (20.0%) of the 13.4 million population were covered by health insurance schemes in 2012, and of those 1.60 million (59.7%) were registered under health financial protection schemes for formal sector employees.^7^^–^^9^ Vulnerable populations, such as children younger than 5 years of age and individuals older than 60 years of age, were eligible for one of the free health-care initiatives, which the government fully subsidize. However, people uninsured, including workers in the informal sector, unemployed individuals, and those living in rural areas, were at high risk of catastrophic health expenditure.

This situation led the Senegalese government to refocus their priorities in realizing UHC, by focusing on improving the quality of health-care provision, strengthening the health workforce and protecting its citizens from catastrophic health expenditure. The new priorities considered the strong political commitment made by the president in 2013, setting equity as a fundamental element in improving access to health care and reducing poverty.^10^^–^^13^ To increase financial protection, the Senegalese government launched a programme for universal health financial protection called *Couverture Maladie Universelle*, in 2013. Following national consultations on health and social actions, the government decided that the programme should: (i) develop basic health financial protection through a community-based health insurance scheme; (ii) reform the compulsory health insurance for formal sector employees; and (iii) strengthen and rationalize the free health-care initiatives. In 2015, the government established a national agency, *Agence de la Couverture Maladie Universelle*, which aims to extend health financial protection coverage to all citizens.

**Fig. 1:**
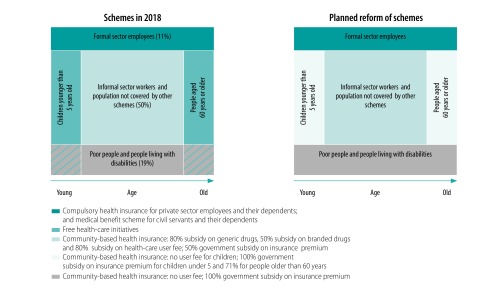
Current and planned financial protection schemes by target population, Senegal

Notes: The percentage in the panel showing schemes in 2018 represents the estimated target population of each scheme out of total population. In the same panel, children younger under 5 years of age and people 60 years of age or older in the poorest income bracket or if they are living with disabilities, are covered by both community-based organizations and the free health-care initiatives.

**Fig. 2:**
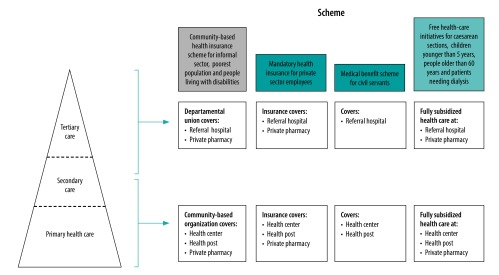
Health care provision and financial protection schemes, Senegal, 2019

Notes: For the mandatory health insurance for private sector employees, services are only covered at health facilities, which employers contracted with. Private pharmacy is not covered in the civil servant scheme, civil servants can instead have a voluntary insurance scheme to cover pharmacy costs. For children younger than 5 years of age, only essential health-care services are covered and hence the benefit package is less attractive than the community-based health insurance scheme.

In 2018, there were three types of financial protection schemes in the country: schemes for formal sector employees; free health-care initiatives; and the health insurance scheme in which community-based health insurance was chosen as the major approach to reach informal sector and rural areas (Fig. 1). All schemes cover health-care provision in all three levels of the health system (Fig. 2).

**Fig. 3:**
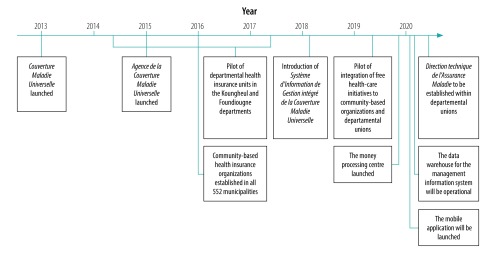
Timeline of the development and progress of the community-based insurance scheme, Senegal, 2013–2020

To advance UHC in Senegal, the coverage of the community-based health insurance scheme needs to expand, and the efficiency of the scheme’s management and procedures needs to improve. Here we discuss the current progress and plans for future development of the community-based health insurance scheme (Fig. 3).

## Community-based insurance scheme

To guarantee financial access to health care for those who were not eligible for the existing schemes, *Couverture Maladie Universelle* established a community-based health insurance scheme consisting of community-based organizations. By the end of 2016, the programme had created 676 organizations in all 552 municipalities, covering the entire country.

These non-profit community-based organizations are operated by part-time community volunteers. Examples of the volunteers’ task include registration of beneficiaries, awareness-raising activities about the scheme, reviewing invoices and reimburse health posts, health centres and private pharmacies for services used by members. The organizations are grouped into departmental unions and these unions oversee the financial coverage for services offered in referral-based hospitals at departmental, regional and national levels (Fig. 2).

Table 1 compares some features of the Senegalese scheme to traditional community-based health insurances in Africa.^14^^,^^15^ The Senegalese scheme can be categorized as community-based health insurance because it is managed by non-profit community organizations and its enrolment is voluntary. However, this scheme differs in several aspects from the traditional schemes. First, the benefit package and insurance premium, together with other insurance management rules, are standardized by the national agency. Second, the Senegalese government fully subsidizes the insurance premium for poor households, identified by the National Family Security Grant programme and people living with disabilities. The premium for all the other enrolees are 50% subsidized. Third, in the scheme, the organizations are handling the insurance premium for health services and drugs that are provided or prescribed by health posts and health centres (primary and secondary health care level). The rest of the fund is pooled at departmental unions for health services and drugs that are provided or prescribed by referral hospitals (tertiary health care). As such, at least for tertiary care, larger financial risks can be pooled by larger groups of people than each community-based organization (Fig. 2).

**Table 1:** Comparison of the Senegalese community-based health insurance and traditional community-based health insurance

Feature	Community-based health insurance in Senegal	Traditional community-based health insurance
Insurers	Non-profit community organizations	Non-profit community organizations
Enrolment	Voluntary	Voluntary
Benefit package	National standard	Not standard
Insurance premium	National standard	Not standard
Regulations	Uniform regulations are set, and the national agency monitor compliance	Not standard
Government subsidy	100% government subsidy to insurance premium of the poor, the persons with disabilities and schoolchildren, and 50% subsidy to all other enrolees	No subsidy by the government
Fund pooling	Two-level pools: a pool for primary and secondary health care benefits managed at community level by community-based organizations, and another pool for hospital care benefits managed at department level by departmental unions of community-based organizations	One pool per community-based health insurance

The health ministry authorizes the community-based organizations to operate under the regulation for social insurance organizations (Regulation no. 07/2009/CM /UEMOA),^16^ which was stipulated by the Western African Economic and Monetary Union. The union sets rules for microinsurance, including community-based organizations, in the light of social mutuality within the union. In particular, community-based organizations should be not for profit.

Household heads can enrol in an organization in the community of residence on behalf of his/her household and is responsible for enrolling all the household members in the same organization. The premium contribution is 7000 Western African CFA Francs (about 12 United States dollars) per person and year, of which 3500 Western African CFA Francs is paid by the member. For poor households and people living with disabilities, the government subsidize user-fees in addition to the premium.

Since the launch of the financial protection programme in 2013, the proportion of people covered by an insurance has increased. According to estimates, the population coverage for all schemes increased from 20.0% in 2012 to 49.6% (7 806 797/15 726 037) in 2018. During the same period, the number of beneficiaries covered by a community-based health insurance increased from 421 670 to 3 000 837.^17^

Many community-based organizations face challenges in the management and operational capacities. A 2017 survey of community-based organizations in Thiès, Diourbel and Tambacounda regions found that of the 2 084 630 eligible non-poor, informal sector households, only 101 187 households (4.8%) had been enrolled in an organization. This low enrolment implies inadequate risk pooling that could threaten the financial sustainability of the community-based organization, particularly in rural areas.^18^ The survey also revealed limitations in the operational capacity, including establishing agreements with local health facilities, reviewing invoices, paying facilities and issues due to the voluntary nature of the work.^18^ Low portability of the benefit package is another challenge, because community-based organizations only cover health care that is provided at local health facilities which they have an agreement with. Finally, the country’s financial protection schemes are fragmented, causing duplication of and inefficiency in the operational procedures of the community-based organizations (Fig. 1). For example, the national agency subsidizes the insurance premium under the community-based health insurance scheme, whereas the agency directly transfers the health-care cost to health facilities under the free health-care initiatives.

## Addressing the challenges

To overcome the challenges the community-based health insurance scheme is facing and to accelerate UHC in the country, the national agency is adopting two major strategic reforms. The first reform relates to several institutional reorganizations of the existing financial protection systems, such as raising risk pooling from community to departmental level and integrating the free health-care initiatives into the community-based scheme. The second reform is the development and implementation of an integrated management information system for more efficient and effective operations of the community-based organizations, as well as monitoring and evaluation of progress towards universal health coverage.

### Institutional changes

#### Raising the risk pool

A key challenge of the community-based scheme is the limited ability for risk pooling, because a large part of the insurance premium remains to be pooled at community level. In addition, each organization has a limited budget to manage the insurance. By moving some of the community-based organizations’ tasks, such as review of invoices, to the departmental union, decision-makers are expecting the scheme to be more efficient through the scale of economy. To ensure sustained community engagement, other functions, such as registration of beneficiaries and awareness campaigns remain at the community level.

Reforming the community-based health insurance scheme by transferring the risk pooling and a part of the management tasks from the community to the department level is necessary for the sustainability of the scheme. Such a reform is planned to follow a pilot project, which was implemented in Koungheul and Foundiougne departments from 2014 to 2017, where only one organization was established in each department instead of each community. These departmental health insurance units, called *l’Unité Départementale d'Assurance Maladie*, were developed as part of an official development assistance project by the Belgian government, in partnership with the Senegalese health ministry.^19^^,^^20^ When the pilot project started in 2012, the aim was to increase the quality of health services by providing health facilities with training and medical equipment. In 2014, the project team expanded the aim of the project by offering financial protection, therefore, one departmental health insurance unit was established in each department to cover 318 640 people in the departments.^19^

The departmental health insurance units are authorized under the same regulations as the community-based organizations. Due to the larger risk pooling, these units are more financially viable to facilitate major interventions in the management and operation of the insurance scheme. First, the units have paid employees, including a director, an accounting manager, a clinical advisor and an administrative assistant. In addition, four staff members are employed to collect premium contributions from the beneficiaries. Second, to increase engagement by local governments, the board of directors includes representatives from the local authority. Third, the units use a special village membership enrolment approach, in which a volunteer is designated by each community to identify all the beneficiaries, to calculate the amount to be collected, so that focal points can collect membership fees and premium contributions based on the information gathered by the volunteer. Fourth, the units apply a discount on the insurance premium for group enrolment. During the implementation of the units, it was estimated that the enrolment rate rose from 2.4% to 25.0%.^19^ The departmental health insurance units continue their activities as a professional and viable health insurance schemes after the end of the pilot project in 2017.

Given these findings, the national agency plans to integrate a part of the functions of community-based organizations into their departmental unions. This integration will reinforce the function of the unions, so that the community-based insurances are controlled and managed at the departmental level. In 2020, the agency plans to set up a directorate, called *Direction Technique de l’Assurance Maladie*, within each departmental union across the country. This directorate will control and manage the operation of the insurance scheme, including invoice review, reimbursement to health facilities, and monitoring of community-based organizations.

#### Integration of the initiatives

The free health-care initiatives are having issues with financial sustainability and efficient use of resources. For example, the initiatives cannot control if health facilities are double claiming health-care expenses for people who both are eligible for an initiative and are having a health insurance. New reforms are under way to transfer the operation and financial responsibility of the free health-care initiatives for caesarean section and health care for children younger than 5 years of age and adults 60 years of age or older to community-based organizations and departmental unions.

Fig. 1 (right panel) shows the planned coverage scheme. In the new scheme, most of the services under the free health-care initiatives will only be offered free of charge to eligible people if they are also enrolled in the community-based insurance scheme. In partnership with the World Bank and United States Agency for International Development, the national agency has piloted the integration of free health-care initiatives into community-based organizations and departmental unions since the first quarter of 2019 in the Kaffrine health district. Many beneficiaries of the free health-care initiatives have already joined community-based organizations. For example, 74.7% (35 000/46 852) of children younger than 5 years of age have been registered to community-based organizations after a door-to-door children census. During the census, surveyors informed caregivers about the community-based insurance scheme and the reform of the free health-care initiatives.

With the integration of the different schemes, the national agency aims to achieve: (i) defragmentation of health service purchasing mechanisms; (ii) unification of financial flows and pooling resources; (iii) increased coverage of the community-based health insurance scheme; (iv) improved relations between health service providers and community-based organizations as well as departmental unions; and (v) improved identification of the targeted beneficiaries for awareness campaigns and collection of fees and premium contributions. Furthermore, since the benefit package of the community-based health insurance scheme is generally larger than that of the free health-care initiatives, the new scheme will entail a package that is more complete and attractive than previous package. The new package covers most eligible services at all levels of the health system including medicines from private pharmacies, and neonatal emergencies care in referral hospitals, which is currently covered by the community-based health insurance scheme, but not by the free health-care initiatives.

### Integrated information system

Currently, most community-based organizations use paper-based records for enrolment and financial accounts, only a few use computers. To facilitate the monitoring of beneficiaries and to provide online payment options, the national agency introduced an integrated management information system, called *Système d’Information de Gestion Intégré de la Couverture Maladie Universelle,* in 2018. The introduction of the system was supported by the World Bank, *Agence Française de Développement* and Japan International Cooperation Agency. The system has seven modules: (i) biometric identification and management of beneficiaries; (ii) a money processing centre for collection of premiums and other funding; (iii) a data warehouse; (iv) registration and monitoring of the beneficiaries; (v) information management for payments and bills; (vi) information management for insurance operation; and (vii) a mobile phone application for beneficiaries. Here we describe the four main modules.

The money processing centre, also called “SUNUCMU”, was launched by the former Prime Minister in April 2019. This module is an electronic platform for collection of subscription fees and premium contributions for community-based organizations, as well as a platform for beneficiary sponsorship and fundraising. Furthermore, the module offers a savings account where the users can save money for their insurance premium.

To reduce the inaccurate data entry and inefficient data processing due to paper-based records, the information system includes a module for registration and monitoring of the beneficiaries called *Système d’Information de Suivi des Mutuelles*. This module enables regular and continuous monitoring of the data entered by community-based organizations, departmental unions and the national agency. The module is now fully synchronized with a module called *Système de Gestion de l’Assurance Maladie*, which manages information relating to the insurance operation, including beneficiaries list, health services used, subsidy from the government and accounting of community-based organizations. 

The mobile phone application “SAMACMU”, which is planned to be launched in early 2020, provides the public with information about the insurance scheme and general health, and it enables patients to find health facilities, make appointments and settle payments. Community-based organizations can also use the application to run awareness campaigns about the insurance scheme.

Finally, the data warehouse will be one of the major evolutions of this reform, since the warehouse will centralize information from the existing various health financial protection schemes. This centralization of data is expected to enhance the monitoring of the schemes and contribute to defragmentation of those schemes. The data warehouse module will be effective from the first quarter of 2020.

## Discussion

The Senegalese government has made a decisive commitment to invest in financial protection through the development of the community-based health insurance scheme. The government foresees that the institutional reforms and the new information management system will improve the insurance management and the efficient use of available resources. However, improvements are still needed, such as increasing the risk pool using a mandatory scheme, reducing fragmentation of schemes, strengthening governance and securing funds to achieve and sustain UHC. 

The survey on community-based organizations in three regions revealed that only a modest proportion of informal-sector households were covered by a community-based health insurance, which is jeopardizing the financial sustainability of the scheme.^18^ Studies from several countries have shown that insurances are offered on a voluntary basis. Eligible households can purposefully select their household members to be insured and pay the insurance premium only for them.^21^^–^^25^ This adverse selection could lead to the situation that the people insured are more likely to be in need of health care. Therefore, a key target in the *National health financing strategy to move towards universal health coverage* is to make enrolment mandatory and the health financing system more centralized in Senegal.^26^ In Africa, the governments in Ghana and Rwanda have introduced such mandatory schemes.^27^^–^^29^ The Senegalese government has opted for an approach to gradually progress towards a mandatory scheme, for example by requiring new members of professional guilds to enrol in a community-based health organization.^30^ Another issue is that public sector engagement to expand the insurance coverage is still weak in the country. In Rwanda, achieving a high coverage rate is one of the performance indicators of local governments, incentivizing the public sector to be actively involved in the insurance scheme.^28^^,^^31^

The on-going reform in Senegal of transferring the risk pooling from community level to department level is expected to reduce fragmentation of the health financing system.^32^^–^^35^ The integration of the free health-care initiatives into the community-based health insurance scheme, should also contribute to reduce fragmentation of the health financial protection schemes. However, in the current health financing system, there is no scheme for risk adjustment beyond the departmental level. The evidence of urban-rural disparity in the financial capacity is potentially threatening the sustainability of the system, particularly in the rural departments.^18^ The integrated management information system should help the national agency to efficiently monitor such a disparity across departments as well as health services that are covered by the different health financial protection schemes. The data generated by monitoring the disparities could be used in the discussion towards risk adjustment and pooling at a higher, national level, achieving a more efficient and equitable system. The progress of implementing the integrated management information system in rural areas is of a concern, since these areas often lack the information technology infrastructure needed. Furthermore, the uptake of this system might be hampered among people with inadequate access to smartphones and/or internet.

The intention of the reforms is to maintain the active engagement and ownership of the community-based health insurance at the community level, while transitioning some financial functions of the scheme to the departmental level. Studies have shown the importance of community engagement and ownership in running an insurance scheme.^27^^,^^28^^,^^34^ In Senegal, the municipalities are still responsible for functions such as registration of beneficiaries and awareness-raising activities about the community-based organizations. However, establishing a governance system in which the voice of the community can be heard and reflected in decisions at the departmental level is still needed. For example, by including more community representatives on the board of the departmental unions, 

Raising more general revenue and increasing the budget for UHC are key strategies for the Senegalese government to sustain full premium subsidy for the vulnerable populations. The cost of implementation and maintenance of the integrated management information system is also of financial concern. Dialogue between the national agency and the finance ministry has started to explore the fiscal space for the community-based health insurance scheme, including implementing tax on sugar-sweetened beverages and introducing social value added tax earmarked for government health-care expenditure.^36^^,^^37^ Such ways of financing UHC may be necessary to overcome the fiscal difficulties to achieve and sustain UHC in Senegal. 
